# Macroscopic comparison of open Metzenbaum and ultrasound‐guided fasciotomy techniques for surgical treatment of the hindlimb proximal suspensory ligament desmopathy in horses: A cadaveric anatomical study

**DOI:** 10.1111/vsu.70102

**Published:** 2026-03-26

**Authors:** Grigorios Maleas, Kristyna Hargitaiova

**Affiliations:** ^1^ Equuria Orthopedics GbR Emstek Germany; ^2^ Department of Biomedical Sciences Cornell University Ithaca New York USA

## Abstract

**Objective:**

To macroscopically characterize and compare the open Metzenbaum (FOM) and ultrasound‐guided (FUG) plantar fasciotomy techniques for decompression of the hindlimb proximal suspensory ligament (PSL), and to determine whether either approach induces intraligamentous splitting (ILS).

**Study design:**

Cadaveric experimental study.

**Sample population:**

Paired hindlimbs from 10 adult horses with no history of hindlimb lameness.

**Methods:**

Each horse contributed one limb to FOM and the contralateral to FUG. All procedures were performed by a single surgeon, and incision measurements were obtained by a blinded examiner. Skin, fascia, and ILS lengths were recorded after dissection. Integrity of the deep branch of the lateral plantar nerve (DBLPN) was evaluated macroscopically. Paired *t*‐tests compared FOM and FUG values (*p* < .05).

**Results:**

Both techniques induced PSL ILS. Mean ± SD incision lengths (cm) for FOM versus FUG were: skin 5.02 ± 0.41 versus 1.28 ± 0.18; fascia 5.54 ± 0.44 versus 4.39 ± 0.22; and ILS 5.33 ± 0.42 versus 4.37 ± 0.24. The FUG technique produced 74% shorter skin, 21% shorter fasciotomy, and 18% shorter ILS incisions compared to FOM (all *p* < .0001). No macroscopic evidence of DBLPN injury was observed.

**Conclusion:**

Both FOM and FUG plantar fasciotomy techniques produced ILS while achieving PSL decompression. FUG required smaller incisions, confirming comparable anatomical efficacy with reduced invasiveness.

**Clinical relevance:**

Intraligamentous splitting appears unavoidable during plantar fasciotomy, but the FUG technique limits its extent and associated soft‐tissue disruption, supporting its use as a safer, less invasive alternative in clinical cases of chronic PSL desmopathy.

## 1 INTRODUCTION

Proximal suspensory ligament (PSL) desmopathy is a frequent cause of chronic hindlimb lameness in sport horses, often leading to performance limitation or early retirement.^1^ The PSL functions as a critical load‐sharing structure that stabilizes the tarsometatarsal and metatarsophalangeal joints during propulsion. Repetitive overstrain, particularly in horses with poor limb conformation or intensive training regimens, can induce microdamage and subsequent fibro‐osseous enthesopathy at the ligament's origin.^2^, ^3^ Chronic cases are commonly complicated by periligamentous fibrosis and enlargement of the PSL within the constraints of the deep plantar fascia, resulting in increased intracompartmental pressure a syndrome comparable to chronic exertional compartment syndrome in humans.^4^, ^5^, ^6^

Conservative management (rest, extracorporeal shockwave therapy, intralesional injections of anti‐inflammatory or regenerative agents) may relieve early inflammatory lesions but is often unsuccessful once chronic compartmental constriction develops.^7^, ^8^ Consequently, surgical decompression via plantar fasciotomy, often combined with neurectomy of the deep branch of the lateral plantar nerve (DBLPN), has become the standard treatment for refractory hindlimb PSL desmopathy.^9^, ^10^ The traditional open Metzenbaum fasciotomy (FOM) is described as a release of the deep plantar fascia to decompress the ligament.^9^, ^10^ However, iatrogenic trauma to the PSL during fasciotomy is a recognized complication, particularly when Metzenbaum scissors are used. Sidhu et al.^11^ performed an ex vivo comparison of Metzenbaum scissors and Y‐shaped fasciotome for deep metatarsal fasciotomy and reported iatrogenic PSL injury in nine and six out of 10 specimens with both instruments, respectively. The injury most often occurred in the distal third of the incision and was deeper with scissors. The authors concluded that Metzenbaum scissors are more likely to cause intraligamentous splitting (ILS) or trauma to the PSL than a fasciotome and recommended the latter to minimize this risk, albeit over 50% of PSLs were also injured with a fasciotome.^11^

Consistent with this, postoperative ultrasonographic findings of hypoechoic intraligamentous changes—interpreted as fiber disruption or splitting induced by the surgical technique—have been reported in horses after fasciotomy.^12^, ^13^ Together, these findings suggest that standard open fasciotomy may unintentionally result in ILS of the PSL, as demonstrated by both imaging and gross pathology in experimental and clinical models.^11^, ^12^, ^13^

At the same time, FOM requires a relatively large skin and fascial incision for exposure, which may predispose to postoperative wound complications such as seroma formation, delayed healing, or cosmetic swelling.^10^, ^14^ To minimize tissue trauma, ultrasound guidance has been introduced as an adjunct to PSL surgery.^12^ By enabling intraligamentous visualization, ultrasound allows the surgeon to perform targeted fasciotomy through small portals, analogous to the evolution of minimally invasive and endoscopic fasciotomy techniques in human orthopedic surgery.^15^, ^16^, ^17^, ^18^ Clinical reports suggest that ultrasound‐guided fasciotomy achieves favorable outcomes while reducing surgical morbidity, but no study has yet provided an anatomical, quantitative comparison of the two techniques.^3^, ^19^

Therefore, this cadaveric study aimed to (1) characterize the anatomical effects of both open and ultrasound‐guided PSL fasciotomy and (2) quantitatively compare the extent of skin, fascial, and ligament incisions between the two methods. We hypothesized that both techniques induce ILS of the PSL, but that the ultrasound‐guided approach achieves comparable decompression through significantly smaller and more targeted incisions.

## 2 MATERIALS AND METHODS

### 2.1 Specimens

A total of 10 horses with no history of hindlimb lameness were included. Causes of euthanasia were diverse and unrelated to the suspensory apparatus (colic, *n* = 7; displaced radial fracture, *n* = 1; pneumonia, *n* = 1; colitis, *n* = 1). Owner's consent was obtained for the use of cadaveric limbs for research purposes. All limbs were harvested within 2 h post‐euthanasia and stored at 4°C until dissection.

### 2.2 Ethical statement

This study used cadaveric material obtained post‐mortem from horses euthanized for clinical reasons and not for research purposes. The ethical framework was extrapolated from the *AAVMC Guidelines for the Use of Animals in Veterinary Education* (2023),^20^ specifically Section 3, which address the use of cadavers and cadaveric tissues. In accordance with these principles and with owner consent obtained, no additional institutional ethical board approval was required.

### 2.3 Experimental design

Each horse contributed one hindlimb to each treatment group: the open fasciotomy (FOM) and the ultrasound‐guided fasciotomy (FUG), yielding *n* = 10 limbs per technique (10 left hindlimbs and 10 right hindlimbs either FOM or FUG). This sample size was selected based on precedent in a comparable cadaveric fasciotomy study and practical feasibility for a controlled technique‐comparison investigation.^11^ Limb allocation was randomized by an independent technician using a coin‐flip method to assign each side to either FOM or FUG.

**FIGURE 1:**
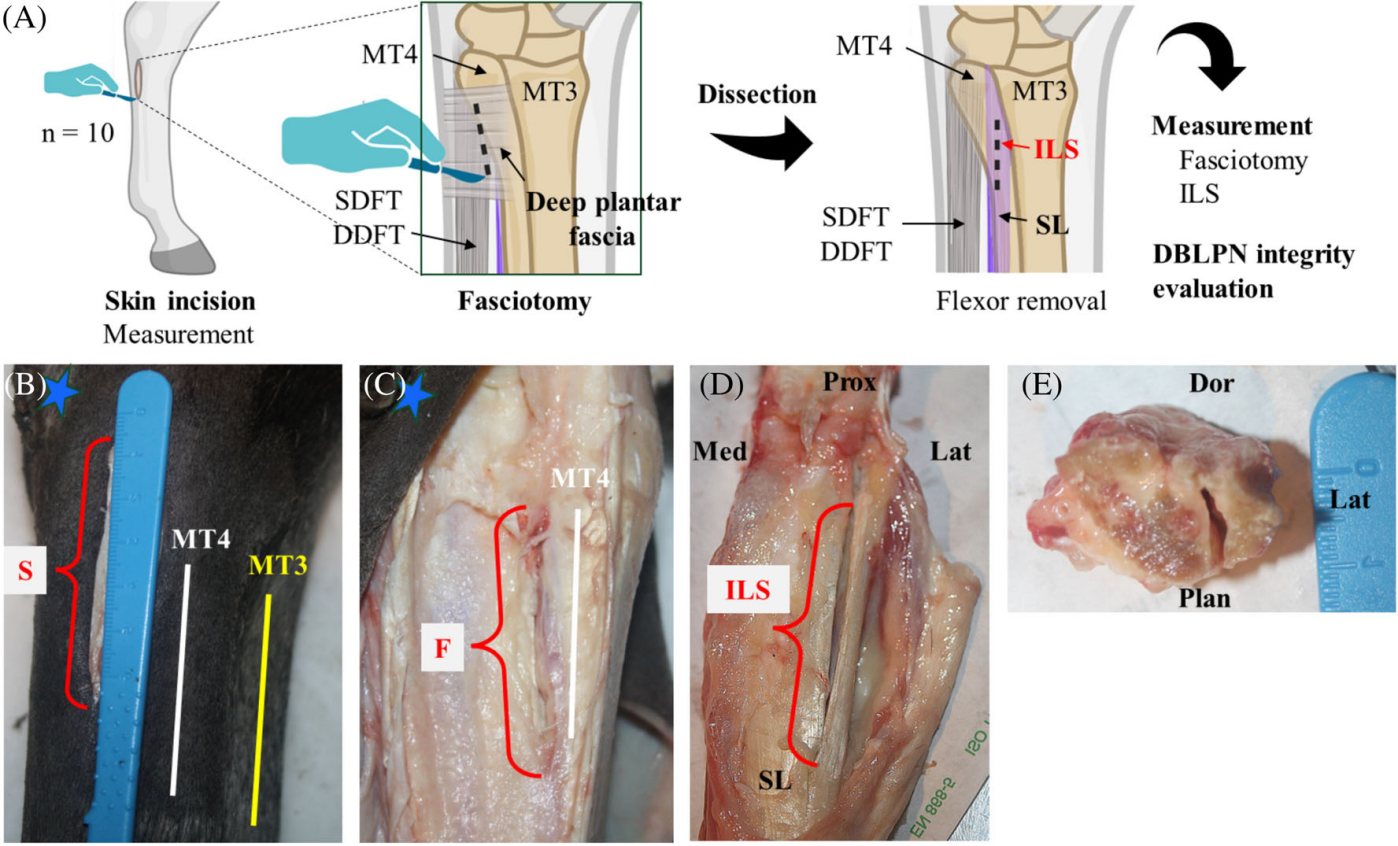
Schematic workflow and gross anatomy of open (FOM) fasciotomy technique. (A) Schematic representation and (B, C) corresponding dissection images showing incision placement plantar to MT4, (D) exposure of the proximal suspensory ligament (PSL) after fascia release, and (E) isolated PSL specimen 2 cm from proximal SL attachment. Blue star, chestnut; DDFT, deep digital flexor tendon; Dor, dorsal; F, fasciotomy; ILS, intraligamentous splitting of the PSL; Lat, lateral; Med, medial; MT3, third metatarsal bone; MT4, fourth metatarsal bone; Plan, plantar; Prox, proximal; S, skin; SDFT, superficial digital flexor tendon; SL, suspensory ligament.

**FIGURE 2:**
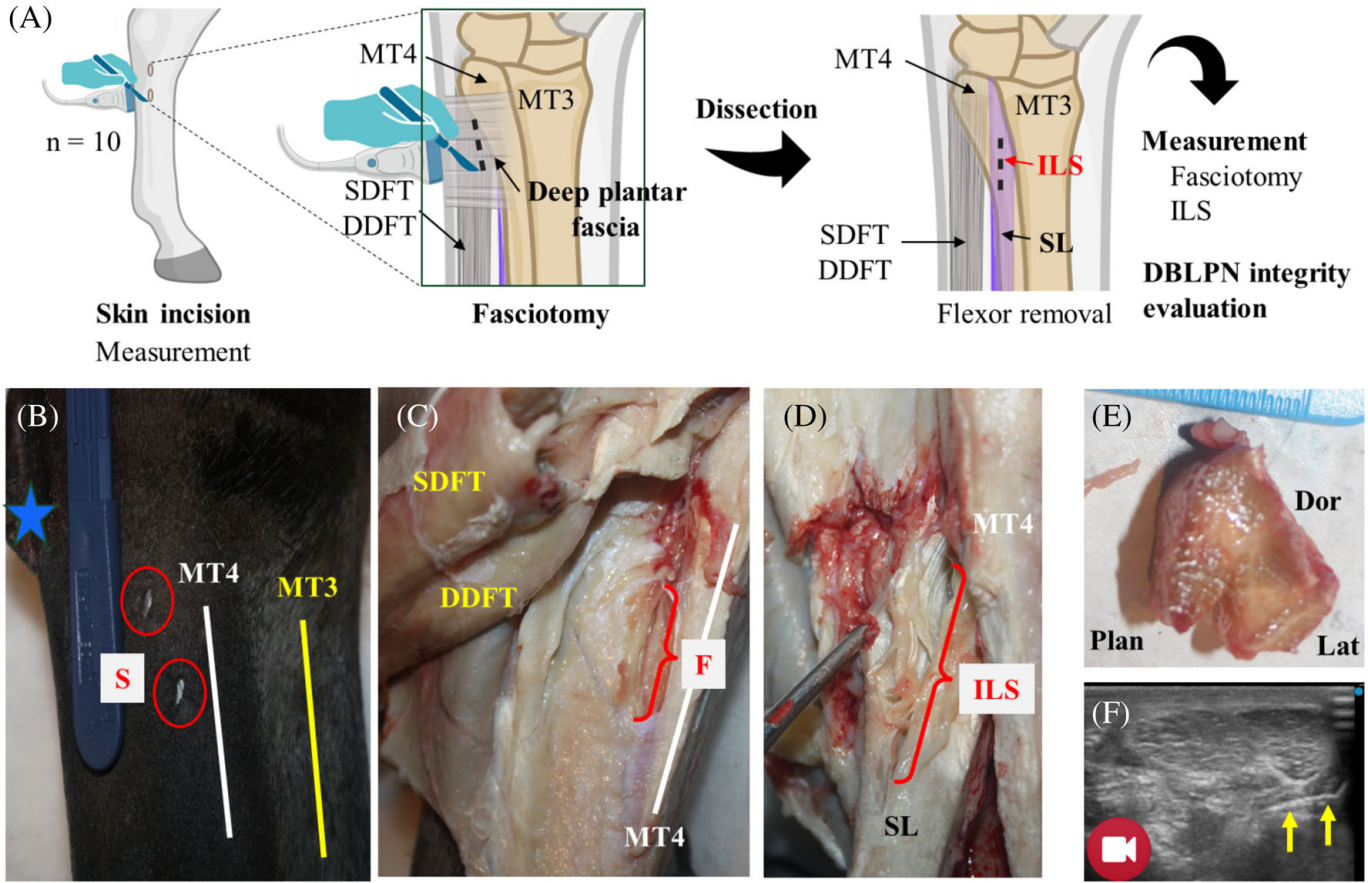
Schematic workflow and gross anatomy of ultrasound‐guided (FUG) fasciotomy technique. (A) Schematic representation and corresponding dissection images showing two small portals under (B) ultrasonographic guidance, (C) fasciotomy incisions, (D) ILS and (E) isolated proximal suspensory ligament (PSL) specimen 2 cm from proximal SL attachment. (F) Video S1 of FUG is attached to this study; yellow arrows = scalpel blade. Blue star = chestnut; DDFT, deep digital flexor tendon; Dor, dorsal; F, fasciotomy; ILS, intraligamentous splitting of the PSL; Lat, lateral; Med, medial; MT3, third metatarsal bone; MT4, fourth metatarsal bone; Plan, plantar; Prox, proximal; S, skin; SDFT, superficial digital flexor tendon; SL, suspensory ligament.

All surgical procedures were performed by a single experienced equine surgeon to minimize interoperator variability. Postoperative macroscopic measurements of incision and ILS lengths were performed by a trained technician blinded to the study objectives to reduce bias. The workflow and anatomical orientation of both approaches are illustrated in Figures 1 and 2.

### 2.4 Surgical techniques

#### 2.4.1 Open fasciotomy (FOM)

A 5–6 cm longitudinal skin incision was made between the fourth metatarsal bone (MT4) and the lateral margin of the superficial digital flexor tendon (SDFT) (Figure 1A,B).^9^ The superficial fascia was sharply incised, and Gelpi retractors were placed to expose the deep plantar fascia. Using Metzenbaum scissors, the deep fascia was released along the longitudinal axis of the PSL in a proximal‐to‐distal direction, following the contour of MT4 (Figure 1C). Adequate fascial release and compartment decompression were subjectively confirmed by digital palpation. After decompression, the fascia and the PSL were isolated and dissected for gross examination to document the extent of ILS of the PSL (Figure 1C–E).

#### 2.4.2 Ultrasound‐guided fasciotomy (FUG)

The PSL was identified ultrasonographically in a transverse plantaromedial view, deep to the head of MT4 which served as the primary osseous landmark (Figure 2A).^12^ The lateral margin of the SDFT and the plantar surface of MT4 were used as consistent soft‐tissue and bony reference points to define the surgical corridor.

A No. 11 scalpel blade was then introduced under real‐time ultrasound guidance approximately 1 cm distal to the head of MT4, positioned between the lateral margin of the SDFT and the plantar surface of MT4 to access the deep plantar fascia and the adjacent PSL (Figure 2F and Video S1). Through this first portal, the deep plantar fascia was incised in a proximal‐to‐distal direction parallel to the longitudinal fiber orientation of the PSL and subsequently in the reverse (distal‐to‐proximal) direction.

A second portal was created approximately 2 cm distal to the first portal, using the same anatomical landmarks, to extend the fascial release over the desired length. Following completion of the procedure, the fascia and the PSL were dissected for gross assessment (Figure 2C–E). Adequacy of fascial release and compartment decompression were similarly confirmed subjectively by digital palpation following completion of the ultrasound‐guided release.

### 2.5 Measurements

Following each procedure: (1) Skin incision length was measured along the external incision line using digital calipers (Figures 1B and 2B). (2) After removal of the skin and flexor tendons, fascia incision length was measured along the released deep plantar fascia (Figures 1C and 2C). (3) The PSL was elevated to visualize the lateral plantar metatarsal nerve, and the extent of ILS was measured along the fiber axis (Figures 1D and 2D). The extent of ILS of the most proximal part of SL was documented (Figures 1E and 2E). (4) Nerve integrity was evaluated macroscopically to confirm the absence of injury to the deep branch of the lateral plantar nerve (DBLPN).

### 2.6 Statistical analysis

All statistical analyses were performed in GraphPad Prism (version 10.6.1 [799]). Data distribution was assessed using the Shapiro–Wilk test, with normality evaluated for paired differences. Given the paired study design and the absence of major deviations from normality, differences between techniques were assessed using paired two‐tailed *t*‐tests. Associations between fasciotomy length and ILS length were assessed using Spearman rank correlation. Effect sizes were quantified using Cohen's *dz* (mean of paired differences divided by the standard deviation of paired differences). Observed statistical power was calculated from the exact *p*‐value of each test using the formula Observed Power = 1 − Φ(*z*₁₋*α*/_2_ − *z*₁₋*p*/_2_), where Φ denotes the standard normal cumulative distribution function, *z*₁₋*α*/_2_ = 1.96 (two‐sided *α* = .05), and *z*₁₋*p*/_2_ is the normal quantile corresponding to the observed *p*‐value. Data are presented as mean ± standard deviation unless otherwise stated. Statistical significance was set at *p* < .05.

All figures and schematic representations of surgical approaches were created using BioRender.com and Microsoft PowerPoint (version 2511).

## 3 RESULTS

### 3.1 Skin incision length

**FIGURE 3:**
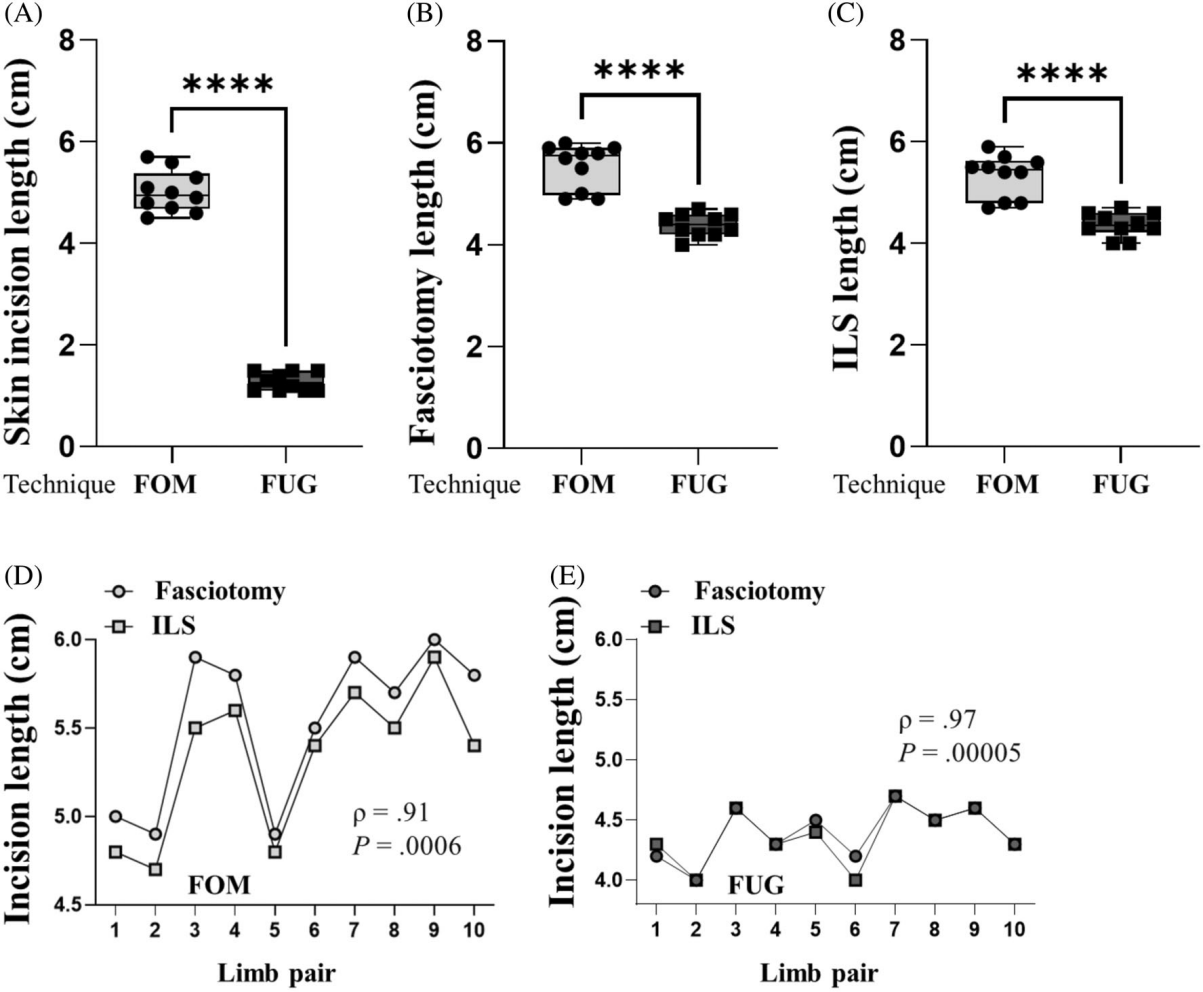
Quantitative comparison of skin incision, fasciotomy and ILS lengths between open (FOM) and ultrasound‐guided (FUG) fasciotomy. (A–C) Box plots showing significantly shorter skin, fascia, and intraligamentous splitting of the PSL (ILS) lengths in FUG compared to FOM (paired *t*‐test, ****p < .0001). ILS was observed in all specimens for both techniques. Individual data points are shown. Bars represent mean ± SD. (D–E) Scatter plots illustrating the association between fasciotomy length and ILS length for (D) FOM and (E) FUG in all specimens, assessed using Spearman rank correlation (*ρ*).

Quantitative outcomes comparing the open (FOM) and ultrasound‐guided (FUG) techniques are summarized in Table 1. The mean skin incision length was 5.02 ± 0.41 cm for the FOM and 1.28 ± 0.18 cm (both portals combined) for the FUG. This corresponded to an FUG/FOM ratio of 25.5%. Thus, the FUG incisions were 74.5% shorter than those in FOM (*p* < .0001, paired *t*‐test; Figure 3A).

**TABLE 1:** Summary of quantitative outcomes for open (FOM) and ultrasound‐guided (FUG) fasciotomy.

Parameter	FOM (mean ± SD, cm)	FUG (mean ± SD, cm)	Mean Δ ± SD	FUG/FOM (%)	FUG reduction vs FOM (%)	*p*‐value	Effect size (dz)	Power (1–*β*)
Skin incision length	5.02 ± 0.41	1.28 ± 0.18	3.74 ± 0.45	25.5	74.5	< .0001	8.40	1.00
Fascia incision length	5.54 ± 0.44	4.39 ± 0.22	1.15 ± 0.35	79.2	20.8	< .0001	3.28	1.00
ILS length	5.33 ± 0.42	4.37 ± 0.24	0.96 ± 0.34	82.0	18.0	< .0001	2.82	1.00

*Note*: Values are presented as mean ± SD (cm). Mean differences (Δ), FUG/FOM ratios, relative reductions (%), *p*‐values, effect sizes (*d*_z_), and achieved statistical power (1–*β*) are shown. FUG produced significantly shorter skin, fascia, and ILS incisions than FOM (*n* = 10 pairs, *p* < .0001 for all comparisons, paired *t*‐test).

Abbreviation: ILS, intraligamentous splitting of the PSL.

### 3.2 Fascia incision length

The mean fascia incision length measured 5.54 ± 0.44 cm for FOM and 4.39 ± 0.22 cm for FUG, representing an FUG/FOM ratio of 79.2% and a 20.8% shorter fascial incision in FUG, with mean difference of 1.15 ± 0.35 cm (*p* < .0001; Figure 3B). Subjective manual assessment confirmed adequate decompression of the proximal suspensory ligament compartment in all specimens following both fasciotomy techniques.

### 3.3 Length of intraligamentous splitting

ILS was observed in all specimens (10/10) following fasciotomy for both techniques. The ILS length averaged 5.33 ± 0.42 cm for FOM and 4.37 ± 0.24 cm for FUG, yielding an FUG/FOM ratio of 82.0% and an incision length reduction of 18.0% in FUG, with a mean difference of 0.96 ± 0.34 (*p* < .0001; Figure 3C). A strong positive association was identified between fasciotomy length and ILS length for both techniques (FOM: Spearman *ρ* = 0.91, *p* = .0006; FUG: Spearman *ρ* = 0.97, *p* = .00005; Figure 3D,E).

### 3.4 DBLPN injury

No macroscopic evidence of injury to the deep branch of the lateral plantar nerve (DBLPN) was observed in either group. The nerve remained anatomically distinct and intact in all specimens (10/10 per group).

### 3.5 Power analysis summary

The calculated effect sizes were *dz* = 8.4 for skin incision, *dz* = 3.28 for fascia incision, and *dz* = 2.82 for ILS length. Observed power, derived directly from the exact *p*‐values using the formula Observed Power = 1 − Φ(*z*₁₋*α*/_2_ − *z*₁₋*p*/_2_), was 0.9999 for skin incision, 0.9969 for fascia incision, and 0.9933 for ILS length, confirming that the study sample size provided full statistical sensitivity to detect the observed differences.

## 4 DISCUSSION

This study provides the first quantitative macroscopic evidence that both the open (FOM) and the ultrasound‐guided (FUG) fasciotomy techniques are associated with longitudinal ILS of the PSL, despite both procedures being historically described as decompressive in nature.^9^, ^10^, ^12^ In all specimens, a longitudinal slit aligned with the PSL fiber axis was identified, indicating that surgical release of the deep plantar fascia may extend into the ligament substance. The FOM, performed with Metzenbaum scissors, produced consistently longer splitting, while FUG induced it to a lesser extent. These findings highlight that longitudinal ILS is a common macroscopic outcome of plantar fasciotomy and should be considered when selecting or interpreting either surgical technique.

In the present study, ILS was observed macroscopically in 100% (10/10) of limbs following FOM. This finding is consistent with, and extends, the work of Sidhu et al.,^11^ who reported ILS in 9/10 limbs using the same instrument. However, while Sidhu et al. primarily quantified the depth of suspensory ligament splitting, the longitudinal extent of ILS was not measured, precluding direct quantitative comparison of splitting length or its relationship to fasciotomy length between studies. Although the two studies differ in their primary outcome measures, together they indicate a high likelihood of ILS when Metzenbaum scissors are used for plantar fasciotomy. This observation is best interpreted as a procedural characteristic rather than an intended therapeutic effect.

Ultrasound‐guided fasciotomy resulted in substantially smaller skin (−74%) and fascia (−21%) incisions, highlighting its minimally invasive profile. Smaller access wounds are associated with less soft‐tissue trauma, lower risk of wound complications (e.g., infection, dehiscence, scar sensitivity), reduced postoperative inflammation and faster recovery, as reported in both equine and human minimally invasive fasciotomy studies.^7^, ^8^, ^14^, ^17^, ^18^, ^21^, ^22^, ^23^ It is important to note that, to date and to the best of the authors' knowledge, there is no clearly established consensus regarding the optimal length of plantar fasciotomy required to achieve adequate decompression of the proximal suspensory ligament. This contrasts with human surgical practice, where minimum release lengths have been defined for certain compartment syndromes, such as carpal tunnel syndrome.^24^

In our study, FUG may have achieved effective compartment release despite a significantly shorter fasciotomy length compared with FOM. Adequacy of decompression was subjectively confirmed by digital palpation at the time of surgery and is consistent with previously reported clinical outcomes following ultrasound‐guided plantar fasciotomy.^3^, ^11^ These findings suggest that extensive fasciotomy length or large skin exposure may not be necessary to achieve decompression of the PSL within its compartment.

Although ILS has not traditionally been regarded as part of plantar fasciotomy, it may represent an unrecognized contributor to the procedure's therapeutic effect. The biological rationale remains incompletely defined, but clinical and ultrasonographic improvements^9^, ^10^ following fasciotomy (an inadvertently ILS) could suggest a reparative response,^12^, ^13^, ^25^ although direct histologic evidence of revascularization and proof of biomechanical restoration are needed. Previous in vitro and in vivo studies have shown that micro‐injury within the medial collateral ligament of the knee during the acute phase of the injury stimulates fibroblast activation, collagen synthesis, and possibly neovascularization—mechanisms analogous to those observed in other connective tissue repair models.^26^, ^27^, ^28^, ^29^, ^30^ Furthermore, fascia has recently been described as a mechanobiological hub and stem‐cell reservoir, capable of initiating regenerative cascades when mechanically disrupted.^25^ Together, these concepts support the view that micro‐injury and decompression could act synergistically in promoting recovery in an acute desmopathy. However, in the absence of clearly defined intraligamentous lesions in chronic PSL desmopathy, the potential benefit of ILS remains questionable and, in the authors' opinion, cannot be recommended.

Building on this rationale, the shorter ILS length observed in FUG (−18%) raises questions about how the extent of ligament splitting may influence this reparative response. While FUG produced a shorter ILS, it achieved sufficient decompression of the PSL—confirmed manually during the procedure—indicating that shorter incisions may still provide adequate anatomical release. Whether a reduced ILS length limits, maintains, or optimizes the biological healing stimulus remains uncertain. In contrast, longer splitting, as consistently seen with FOM, may enhance this mechanical trigger but also increase iatrogenic trauma, particularly in the distal PSL where the fascia‐ligament interface is narrow.^11^ The shorter and more targeted release achieved with FUG may therefore represent a more balanced approach between mechanical stimulus and tissue preservation.

No macroscopic injury to the deep branch of the lateral plantar nerve (DBLPN) was observed in either group, which supports the anatomical safety of both techniques when performed by experienced surgeons. Nonetheless, nerve evaluation in this study was visual only; microscopic or electrophysiologic assessment would be needed to exclude microscopic nerve fiber injury, as reported in related human plantar fascia release procedures.^31^

The power analysis confirmed that the observed differences were statistically robust, with all comparisons achieving an effect size greater than 2.8 and post hoc power of ~1.00, confirming the reliability of the findings achieved with our sample size.

## 5 STUDY LIMITATIONS

This study was performed on cadaveric limbs, and therefore functional parameters such as postoperative inflammation, healing time, and long‐term outcomes could not be evaluated. In addition, all specimens represented clinically normal proximal suspensory ligaments; consequently, macroscopic effects of fasciotomy may differ in chronically diseased ligaments characterized by fibrosis, enlargement, or periligamentous adhesions. Further in vivo validation is warranted to determine whether the reduction in incision size translates to measurable clinical benefits.

The choice of Metzenbaum scissors reflects their widespread availability and routine use in equine surgical practice; however, alternative instruments such as fasciotome‐based systems were not evaluated and may produce different tissue effects. Although no macroscopic injury to the DBLPN was detected, the evaluation was limited to gross inspection. Future work should include microscopic and electrophysiologic analyses to better define neurovascular safety margins, as well as in vivo studies comparing clinical outcomes of different fasciotomy techniques and instruments under physiologic loading conditions. Histological analysis was beyond the scope of this study but is strongly recommended for future investigations aimed at investigating tissue‐level and neurovascular effects of both techniques.

## 6 CONCLUSION

This study provides the first macroscopic evidence that both open Metzenbaum and ultrasound‐guided plantar fasciotomy can be associated with longitudinal intraligamentous splitting of the PSL under the controlled conditions evaluated. The open Metzenbaum technique was associated with longer splitting, whereas the ultrasound‐guided approach produced a shorter, more targeted ILS while achieving adequate decompression with smaller skin and fascial incisions. These findings indicate that ILS may occur during plantar fasciotomy and should be considered when interpreting the mechanical effects of different surgical approaches. Future in vivo studies are warranted to determine how ILS extent and incision size influence ligament healing, postoperative recovery, and clinical outcomes in athletic horses.

## AUTHOR CONTRIBUTIONS

Maleas G, DVM: Conceptualization (lead), investigation (lead), methodology (lead), project administration (supporting), resources (lead), writing – original draft preparation (equal), writing – review and editing (equal). Hargitaiova K, DVM: Data curation (lead), formal analysis (lead), project administration (lead), supervision (lead), validation (lead), visualization (lead), writing – original draft preparation (equal), writing – review and editing (equal).

## CONFLICT OF INTEREST STATEMENT

The authors declare no conflicts of interest.

## Supporting information


Video S1.

## Data Availability

The data that support the findings of this study are available from the corresponding author upon reasonable request.
